# High Skin Sympathetic Nerve Activity in Patients with Recurrent Syncope

**DOI:** 10.3390/jpm11111053

**Published:** 2021-10-21

**Authors:** Tien-Chi Huang, Nai-Yu Chi, Chih-Sung Lan, Chang-Jen Chen, Shih-Jie Jhuo, Tsung-Han Lin, Yi-Hsueh Liu, Li-Fang Chou, Chien-Wei Chang, Wei-Sheng Liao, Pei-Heng Kao, Po-Chao Hsu, Chee-Siong Lee, Yi-Hsiung Lin, Hsiang-Chun Lee, Ye-Hsu Lu, Hsueh-Wei Yen, Tsung-Hsien Lin, Ho-Ming Su, Wen-Ter Lai, Wei-Chung Tsai, Shien-Fong Lin, Chien-Hung Lee

**Affiliations:** 1 Division of Cardiology, Department of Internal Medicine, Kaohsiung Medical University Hospital, Kaohsiung Medical University, Kaohsiung 807, Taiwan; 990314kmuh@gmail.com (T.-C.H.); marchchi@gmail.com (N.-Y.C.); chihsung.lan@gmail.com (C.-S.L.); jhuoshihjie@gmail.com (S.-J.J.); moresque612@yahoo.com.tw (T.-H.L.); liuboy17@gmail.com (Y.-H.L.); 850194@gmail.com (L.-F.C.); achiengogo@gmail.com (C.-W.C.); casablansa198711@gmail.com (W.-S.L.); galaxykao@gmail.com (P.-H.K.); pochao.hsu@gmail.com (P.-C.H.); lcsphk@ms18.hinet.net (C.-S.L.); caminolin@gmail.com (Y.-H.L.); hclee33@gmail.com (H.-C.L.); yehslu@cc.kmu.edu.tw (Y.-H.L.); hweyen@cc.kmu.edu.tw (H.-W.Y.); lth@kmu.edu.tw (T.-H.L.); cobeshm@seed.net.tw (H.-M.S.); wtlai@cc.kmu.edu.tw (W.-T.L.); 2 Division of Cardiology, Department of Internal Medicine, Show Chwan Memorial Hospital, Changhua 500, Taiwan; pipi75710@gmail.com; 3 Department of Internal Medicine, Kaohsiung Municipal Ta-Tung Hospital, Kaohsiung Medical University, Kaohsiung 801, Taiwan; 4 Department of Internal Medicine, Kaohsiung Municipal Siaogang Hospital, Kaohsiung Medical University, Kaohsiung 812, Taiwan; 5 Department of Internal Medicine, Faculty of Medicine, College of Medicine, Kaohsiung Medical University, Kaohsiung 807, Taiwan; 6 Institute of Biomedical Engineering, National Chiao Tung University, Hsinchu 300, Taiwan; shienfong.lin@gmail.com; 7 Department of Public Health, College of Health Science, Kaohsiung Medical University, Kaohsiung 807, Taiwan; cnhung@kmu.edu.tw; 8 Research Center for Environmental Medicine, Kaohsiung Medical University, Kaohsiung 807, Taiwan

**Keywords:** skin sympathetic nerve activity, vasovagal syncope, head-up tilting test, heart rate variability

## Abstract

(1) Background: The autonomic imbalance plays a role in vasovagal syncope (VVS) diagnosed by head-up tilting test (HUT). neuECG is a new method of recording skin electrical signals to simultaneously analyze skin sympathetic nerve activity (SKNA) and electrocardiogram. We hypothesize that SKNA is higher in subjects with tilt-positive than tilt-negative and the SKNA surges before syncope. (2) Methods: We recorded neuECG in 41 subjects who received HUT (according to the “Italian protocol”), including rest, tilt-up, provocation and recovery phases. Data were analyzed to determine the average SKNA (aSKNA, μV) per digitized sample. Electrocardiogram was used to calculate standard deviation of normal-to-normal beat intervals (SDNN). The “SKNA-SDNN index” was calculated by rest aSKNA multiplied by the ratio of tilt-up to rest SDNN. (3) Results: 16 of 41 (39%) subjects developed syncope. The aSKNA at rest phase is significantly higher in the tilt-positive (1.21 ± 0.27 µV) than tilt-negative subjects (1.02 ± 0.29 µV) (*p* = 0.034). There are significant surges and withdraw of aSKNA 30 s before and after syncope (both *p* ≤ 0.006). SKNA-SDNN index is able to predict syncope (*p* < 0.001). (4) Conclusion: Higher SKNA at rest phase is associated with positive HUT. The SKNA-SDNN index is a novel marker to predict syncope during HUT.

## 1. Introduction

Syncope is the abrupt, transient and complete loss of consciousness that results from global hypoperfusion of the brain with spontaneous recovery. Vasovagal syncope(VVS) is the most common cause of syncope and is considered a benign condition [1]. Head-up tilt testing (HUT) is used for the diagnosis in patients with suspected VVS [2], and heart rate variability (HRV) have been used during HUT to analyzed the balance of the autonomic nervous system (ANS) in patients with VVS [3,4]. In a previous study, VVS might be due to the abrupt withdrawal of peripheral sympathetic activation, with a concomitant increase in vagal outflow, resulting in symptomatic hypotension and/or inappropriate bradycardia [5]. However, the disturbance or dysregulation of ANS in VVS is not fully clarified.

Based on the finding of sympathetic innervation of the skin in the thorax, “neuECG”, a novel noninvasive method has been developed to record skin sympathetic nerve activity (SKNA) and estimate sympathetic tone in humans [6]. SKNA was a reliable method to estimate stellate ganglion nerve activity, which represented the cardiac sympathetic tone [7]. Uradu et al. found that SKNA precedes the onset and offset of atrial arrhythmia [8]. Seventy-three percent of ventricular tachycardia episodes were preceded by SKNA in the admitted electrical storm patients [6]. Furthermore, the crescendo SKNA pattern was found to be associated with ventricular arrhythmia [9]. Our study aims to analyze the ANS changing during HUT by the neuECG and hypothesize that sympathetic tone is higher in tilting positive than negative subjects.

## 2. Materials and Methods

### 2.1. Participants

Forty-one subjects (29 females and 12 males) diagnosed with recurrent syncope were enrolled. The subjects received HUT to diagnose VVS. This study was approved by the ethics committee of Kaohsiung Medical University Hospital, and all study subjects provided written informed consent. This study is registered with ClinicalTrials.gov, identifier: NCT03243448. The demographic data and clinical characteristics including diabetes mellitus (DM), hypertension, cerebrovascular accident (CVA), hepatitis B, ventricular tachycardia (VT), atrial fibrillation (AF), chronic obstructive pulmonary disease (COPD), mitral valve prolapse (MVP) and left ventricular ejection fraction (LVEF) by echocardiography were collected if data were available.

### 2.2. Head-Up Tilt Testing

The protocol used for HUT was modified from the “Italian protocol” [10]. In brief, the HUT is divided into four phases with rest, tilt-up, provocation and recovery phase. The HUT started with 10 min baseline recording when the subject was in the supine position (rest phase), then the subject was tilted at 70 degrees for 20 min (tilt-up phase). If syncope did not occur within 20 min after tilt, 300 µg of nitroglycerin was administrated sublingually to the subject. The subject then remained upright for 20 min (provocation phase) and returned to the supine position for 5 min if syncope developed or after a total of 40 min of tilt (recovery phase). Simultaneously electrocardiogram (ECG), blood pressure (BP), neuECG recordings were performed during HUT. The ECG and neuECG were recorded simultaneously and continuously. The heart rate (HR) and BP were recorded at 1~3 min intervals during HUT according to the hemodynamic changes in each phase. If syncope happened, the provocation phase would be early terminated and advance into the recovery phase. The BP and HR would be recorded and averaged to present the hemodynamic status in each phase. A positive result of the HUT was defined as the symptoms of syncope or presyncope associated with bradycardia, sinus pause, hypotension or both.

### 2.3. neuECG

**Figure 1:**
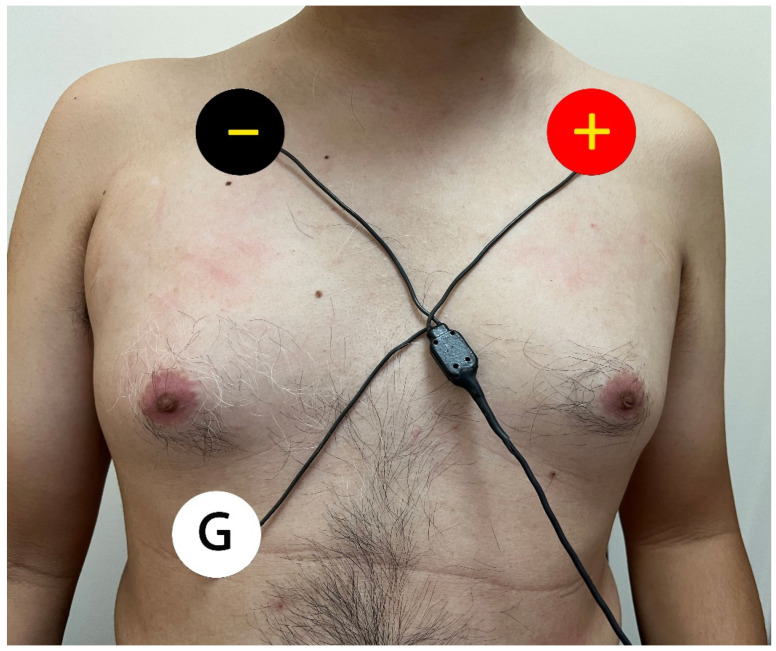
The illustration of electrodes locations. The SKNA is recorded from the negative (black) electrode located in the right subclavicular area to the positive (red) electrode in the left. The ground electrode (white) serves as reference.

The detailed method of neuECG recording was modified from our previous paper [6]. In brief, neuECG used conventional ECG electrodes in lead I configuration and equipment with a very high sampling rate (10,000 Hz) and wide sampling bandwidth (1–2000 Hz) version of MEGA ME6000 Biomonitor System to harvest the electrical signal from the chest wall of research subjects (Figure 1). Then, bandpass filters the signals between 500 to 1000 Hz to display SKNA and between 1 to 150 Hz to display ECG. The neuECG recording was made during rest, tilt-up, provocation and recovery phase in HUT. Data were analyzed to determine the average SKNA (aSKNA, in µV) per digitized sample during the monitoring period. The SKNA was measured around 1–4 pm in each subject. The study participants rested supine for at least 10 min before SKNA measuring in the HUT recording room. The average temperature and moisture were around 23 ± 2 °C and 50 ± 5% in the recording area. The SKNA pattern was analyzed automatically by our customized software and observation of the independent researcher who did not know the subject who was tilt-positive or not.

### 2.4. Heart Rate Variability

We use Matlab-based software heart rate variability analysis software (HRVAS) to analyze the HRV. In brief, the R peak of QRS complex in ECG signal obtained by the neuECG was automatically detected by the modified Pan Tompkins algorithm, and the R-R interval was obtained beat by beat. Time-domain and frequency-domain of HRV were calculated by the Matlab 2013 software and its plug-in “HRVAS” [11]. The standard deviation of normal to normal beat intervals (SDNN) and root mean square of the successive differences (RMSSD) represent the 5 min time-domain HRV. For the frequency-domain HRV, spectral power for HRV was analyzed from the 5 min ECG tracing. The total power (TP), very-low-frequency (VLF; 0.003–0.04 Hz), low-frequency (LF; 0.04–0.15 Hz), high-frequency (HF; 0.15–0.4 Hz) components, low-frequency normalized unit (LFnu), high-frequency normalized unit (HFnu), and LF to HF ratio (LF/HF) were operated based on the frequency—domain analysis. LFnu was calculated as LF/(TP − VLF)*100. HFnu was calculated as HF/(TP − VLF)*100. The “SKNA-SDNN index” defined by rest aSKNA multiplied by the ratio of tilt-up to rest SDNN was used to survey the autonomic interaction in our study.

### 2.5. Statistics

The percentage was used to express the distribution of categorical data. Continuous variables were summarized as mean ± standard deviation if normal distribution and median/Q1/Q3 if non-normal distribution. Independent t-test was used to compare demographic data, clinical characteristics, aSKNA, BP, and HR between the tilt-positive and tile-negative groups. Mann-Whitney U test was used to compare HRV parameters between these two groups because the parameters of HRV were non-normal distribution. Friedman test was used to determine the changes of aSKNA in 4 phases in each group. The Friedman test was also used to compare the aSKNA 120, 90, 60, 30 s before, during and 30, 60, 90, 120 s after syncope in the tilt-positive group. Wilcoxon Signed Ranks Test was used for post-hoc analysis if the Friedman test was significant. Mann-Whitney U test was also used to compare the aSKNA 180, 150, 90, 60 30 s before, during and 30, 60, 90, 120, 150, 180, 210, 240, 270 and 300 s after sublingual nitroglycerin (NTG) was taken. Generalized estimating equation model with an exchangeable correlation structure adjusted for age and gender was used to compare the corresponding BP and HR changes by aSKNA between the tilt-positive and tilt-negative groups. The parameters of SKNA and HRV were used to predict syncope by the receiver operating characteristic (ROC) curve analysis. The optimal cut-point value was the point on the ROC curve nearest to (0, 1). Statistical analysis was performed with Stata 16. A two-tailed *p* value of ≤ 0.05 was considered statistically significant.

## 3. Results

### 3.1. Patient Characteristics

A total of 41 (29 women and 12 men) subjects were included in the study. Sixteen (12 women and 4 men) of 41 (39%) subjects developed syncope and all the syncope was induced by sublingual nitrate provocation. The demographic data and clinical characteristics of the subjects are summarized in Table 1. There is no difference between the tilt-positive and tilt-negative groups regarding age, gender, DM, hypertension, CVA, hepatitis B virus infection, VT, AF, COPD, MVP, and LVEF.

### 3.2. The Comparisons of BP, HR and aSKNA in Different Phases

**Figure 2:**
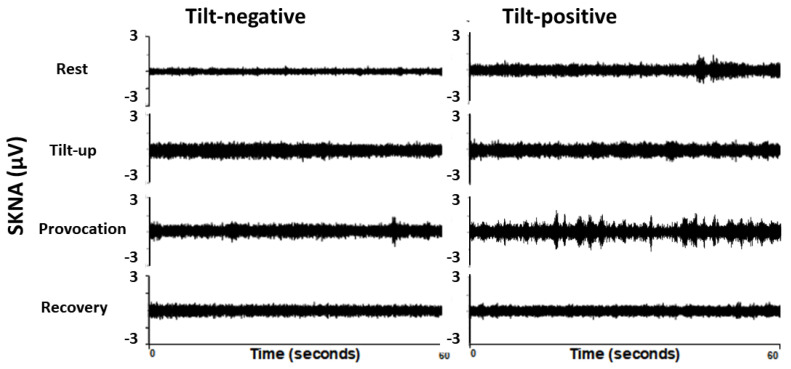
The typical SKNA pattern from a tilt-negative and a tilt-positive subject. The rest phase SKNA is significantly higher in tilt-positive than tilt-negative subjects. The SKNA is non-significantly higher in the tilt-positive than in the tilt-negative subject in the other three phases. SKNA, skin sympathetic nerve activity.

**Figure 3:**
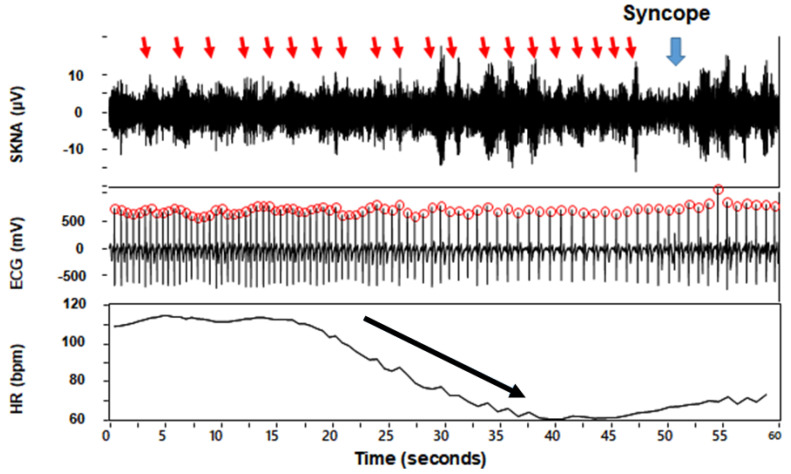
The SKNA pattern of a subject with cardiac inhibitory type VVS. Red arrows indicate the SKNA bursts. The blue arrow indicates the episode of syncope. The bursts of SKNA noticed before the syncope episode are associated with decreasing but not increasing HR in a tilt-positive subject. ECG, electrocardiogram; HR, heart rate; SKNA, skin sympathetic nerve activity; VVS, vasovagal syncope.

**Figure 4:**
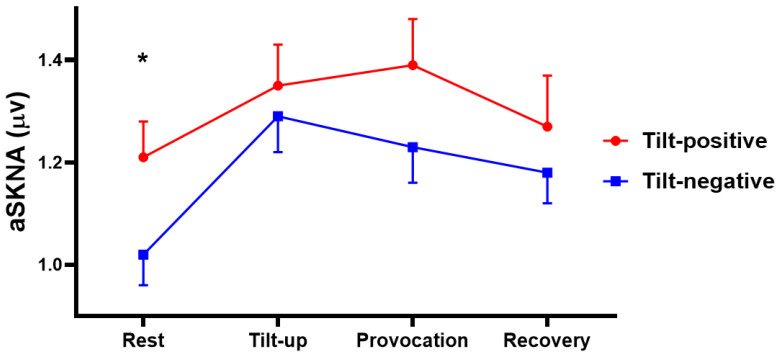
The comparison of aSKNA during HUT between the tilt-positive and negative group. The aSKNA is significantly higher in the tilt-positive than the tilt-negative group at the rest phase. There is a crescendo pattern of aSKNA at tilt up and provocation phases in the tilt-positive group. However, there is a crescendo-decrescendo pattern of aSKNA at tilt up and provocation phases in the tilt-negative group. * *p* < 0.05 when compared with the tilt-negative group. aSKNA, average skin sympathetic nerve activity; HUT, head up tilting.

Table 2 shows the hemodynamic parameters including HR, systolic BP (SBP), diastolic BP (DBP) and aSKNA during the four phases. aSKNA is noted to be higher in the tilt-positive group than the tilt-negative group in the rest phase (1.21 ± 0.27 versus 1.02 ± 0.29 μV, *p* = 0.034), but is no difference in tilt-up, provocation and recovery phases. SBP is lower in the tilt-positive than the tilt-negative group in the provocation phase (*p* = 0.040). HR is also lower in the tilt-positive group than tilt-negative in the recovery phase (*p* = 0.003). We also calculated the SKNA ratio between tilt-up/rest, provocation/rest and provocation/tilt-up to understand the SKNA changes between different phases in both groups. The tilt-up/rest aSKNA ratio means that increasing aSKNA from rest to tilt-up phase is higher in the tilt-negative than the tilt-positive group (1.13 ± 0.18 versus 1.30 ± 0.29, *p* = 0.038). Figure 2 shows the typical SKNA pattern from a tilt-negative and a tilt-positive subject. The rest phase SKNA is increased and has more nerve bursts in tilt-positive than tilt-negative subjects. Figure 3 shows the SKNA pattern of a patient with cardiac inhibitory type VVS. The bursts of SKNA are associated with HR decreasing but not increasing before syncope occurs. Figure 4 demonstrates the comparison of aSKNA between two groups in the four phases. The aSKNA is significantly higher in the tilt-positive than in the tilt-negative group at the rest phase (*p* = 0.034). The aSKNA changes during HUT in both groups (both *p* < 0.001). There is a significant increase of aSKNA from rest to tilt-up phase in both groups (both *p* ≤ 0.018). From tilt-up to provocation phase, the SKNA significantly decreases in the tilt-negative group (*p* = 0.042) but insignificantly increases in the tilt-positive group (*p* = 0.679).

**Figure 5:**
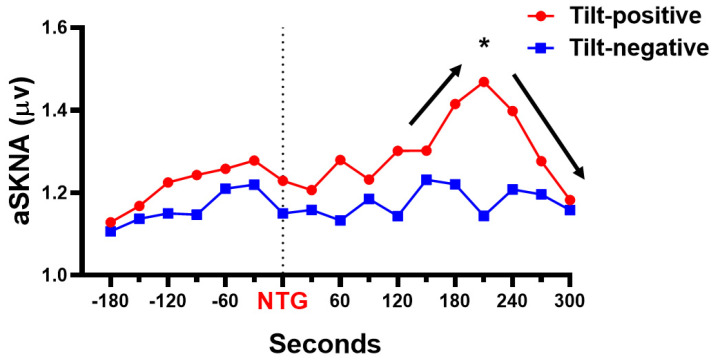
The difference of aSKNA trends after NTG provocation between tilt-positive and negative group. The crescendo-decrescendo pattern (black arrows) of aSKNA is noticed only in the tilt-positive group but not the tilt-negative group after NTG provocation, meaning that the tilt-positive group has an aSKNA surge. The aSKNA is significantly higher at 210 s after NTG administration in the tilt-positive than the tilt-negative group. * *p* < 0.05 when compared with the tilt-negative group. aSKNA, average skin sympathetic nerve activity; NTG, nitroglycerin.

**Figure 6:**
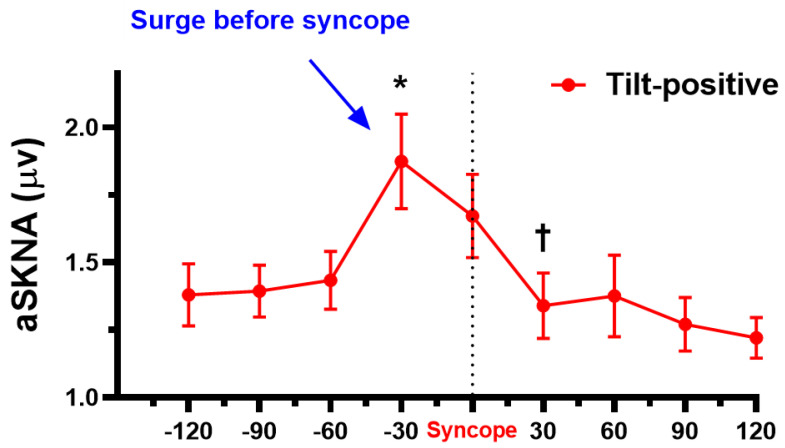
The aSKNA surge before syncope. The aSKNA has significantly changes 120 s before, during and 120 s after syncope in the tilt-positive group (*p* < 0.0001). The surge of aSKNA is noticed before the onset of syncope (−60 s versus −30 s). The aSKNA decreases immediately after aSKNA surge (syncope versus 30 s). * *p* < 0.05 when compared with −60 s. ^†^ *p* < 0.05 when compared with syncope. aSKNA, average skin sympathetic nerve activity.

We also analyze the temporal relationship between SKNA, NTG provocation, and syncope. Figure 5 shows the surge of SKNA after NTG provocation in the tilt-positive but not the tilt-negative group. The SKNA is significantly higher at 210 s after nitroglycerin administration in tilt-positive (1.47 ± 0.43 μV) than in tilt-negative (1.14 ± 0.35 μV) group (*p* = 0.008). The crescendo-decrescendo SKNA pattern in the provocation phase is found in the tilt-positive but not the tilt-negative group. Based on the “crescendo-decrescendo SKNA pattern”, we further analyze the SKNA before and after syncope in the tilt-positive group (Figure 6). The aSKNA is significantly changed 120 s before, during, and 120 s after syncope in the tilt-positive group (*p* < 0.0001). The post-hoc analysis shows that the surge of SKNA is noticed before the onset of syncope (−60 s versus −30 s, *p* = 0.003) and the withdraw of SKNA is found 30 s after syncope immediately (syncope versus 30 s, *p* = 0.006).

### 3.3. The Effect of aSKNA on BP and HR in Different Phases

Fifteen of 41 patients have comprehensive corresponding SBP/DBP/HR in all phases. To evaluate the relationship between sympathetic drive and corresponding hemodynamic changes, we analyze the relationship between BP parameters (including SBP, DBP, mean BP (MBP)), HR, and SKNA in all phases by a generalized estimating equations model (Table 3). In the tilt-positive group, there are positive linear relationships between aSKNA and SBP, between aSKNA and HR at the rest phase, and between aSKNA and DBP at the recovery phase (all *p* ≤ 0.003). In the tilt-negative group, there are positive linear relationships between aSKNA and DBP, between aSKNA and MBP at the tilt-up phase, and between aSKNA and SBP at the recovery phase (all *p* ≤ 0.035). Interestingly, at the provocation phase, the effects of aSKNA on BP and HR are opposite between the tilt-positive and tilt-negative groups. In the tilt-positive group, all BP parameters have negative linear relationships with aSKNA (all *p* ≤ 0.022). In the tilt-negative group, SBP and MBP have positive linear relationships with aSKNA (both *p* ≤ 0.002) and HR has a positive linear relationship with aSKNA (*p* < 0.001). The significantly opposite linear relationship between tilt-positive and tilt-negative group are demonstrated (*p* for the difference in adjusted mean = 0.038, 0.016, 0.026, and <0.001 for SBP, DBP, MBP, and HR, respectively). The above results show the total opposite response of BP parameters and HR to SKNA between tilt-positive and negative groups. The paradoxical hemodynamic response to SKNA is noticed only in the tilt-positive group at the provocation phase.

### 3.4. Heart Rate Variability in Different Phases

The HRV parameters including SDNN, RMSSD, LF/HF, and LFnu in each phase are shown in Table 4. There is no difference regarding HRV parameters between tilt-negative and positive groups in each phase. We also analyze the ratio of SDNN from rest to tilt-up, rest to provocation and tilt-up to provocation to understand the changes of SDNN in different phases in both groups. We find that the tilt-up/rest and provocation/rest ratios of SDNN are significantly higher in the tilt-positive than tilt-negative group (both *p* ≤ 0.02).

### 3.5. Predictors of Syncope

**Figure 7:**
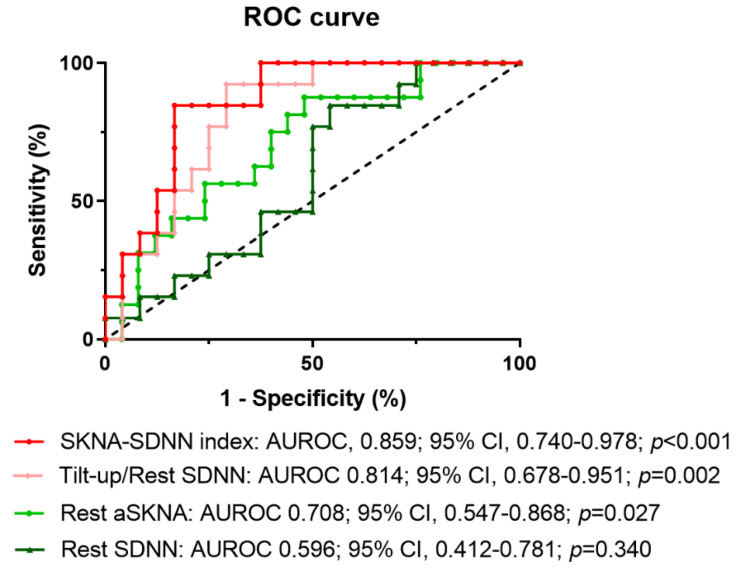
The ROC curve analysis for predicting syncope. Tilt-up/rest SDNN, rest aSKNA and rest SDNN and SKNA-SDNN index are used for ROC curve analysis in predicting syncope. SKNA-SDNN index has the highest value in predicting syncope. aSKNA, average skin sympathetic nerve activity; AUROC, the area under receiver operating characteristic curve; ROC, receiver operating characteristic; SDNN, standard deviation of normal-to-normal beat intervals; SKNA-SDNN index, rest aSKNA multiplied by the ratio of tilt-up to rest SDNN.

Since rest aSKNA and tilt-up/rest SDNN are higher in the tilt-positive group, we use those parameters to predict syncope by ROC analysis. We also use the “SKNA-SDNN index”, the autonomic interaction parameter to predict syncope. Figure 7 shows the ROC curve analysis using different parameters to predict the syncope episode during HUT. The area under ROC curve (AUROC) of “SKNA-SDNN index”, tilt-up/rest SDNN, rest aSKNA and rest SDNN are shown. Although the rest aSKNA and tilt-up/rest SDNN could predict the occurrence of syncope (AUROC = 0.708 and 0.814, respectively, both *p* ≤ 0.027), the “SKNA-SDNN index” has the highest value in predicting syncope (AUROC 0.859, *p* < 0.001). A value of “SKNA-SDNN index” > 1.183 µV is the optimal cut-off point to predict syncope during HUT (sensitivity 85%, specificity 83%).

We have ever performed the sensitivity analysis to compare the possible combinations of SKNA-SDNN parameters for the value of predicting syncope and to test the robustness of the “SKNA-SDNN index”. The AUROC of the possible combinations of SKNA-SDNN parameters including aSKNA*(tilt-up/rest SDNN), aSKNA*(provocation/rest SDNN) and aSKNA*(recovery/rest SDNN) are 0.859 (95% CI: 0.740–0.978), 0.842 (95% CI: 0.706–0.977) and 0.816 (95% CI: 0.665–0.968), respectively. The corresponding optimal cut-off points of those SKNA-SDNN parameters are 1.183 (sensitivity 85%, specificity 83%), 3.330 (sensitivity 73%, specificity 78%), and 1.447 (sensitivity 85%, specificity 74%), respectively. Thus, we chose aSKNA*(tilt-up/rest SDNN) as the “SKNA-SDNN index”.

## 4. Discussion

We demonstrate the novel findings in this study. (1) The SKNA at the rest phase during HUT is higher in the tilt-positive than the tilt-negative group. The rest SKNA can predict the syncope episode. (2) The tilt-up/rest SDNN ratio is higher in the tilt-positive than in the tilt-negative group. The tilt-up/rest SDNN ratio can also predict the syncope episode. (3) Tilt-positive subjects have the paradoxical hemodynamic response to SKNA at the NTG provocation phase. (4) The presyncope surge of SKNA and post-syncope rapid withdrawal of SKNA are noticed. (5) The “SKNA-SDNN index” is useful for predicting syncope (AUROC 0.859, sensitivity 85%, and specificity 83%). Those findings suggest that the sympathetic activity, represented by SKNA, at the rest phase is higher in the tilt-positive than the tilt-negative group. The parasympathetic activation, represented by tilt-up/rest SDNN ratio is also higher in tilt-positive than the tilt-negative group. Meaning that co-activation of sympathetic and parasympathetic tone may be partially responsible for the autonomic dysregulation mechanism of VVS; thus, we can use the “SKNA-SDNN index” to predict syncope in those patients. The paradoxical hemodynamic response to sympathetic stimulation of the tilt-positive subjects may also be a possible mechanism of VVS. The clinical implication of our study is using SKNA combined with HRV parameters to predict the syncope, and it also helps us understand the ANS dysregulation in VVS.

### 4.1. Autonomic Regulation and Syncope

One of the most common etiology of recurrent syncope is the reflex syncope mediated by the ANS regulation [12]. The measurements of ANS by HRV showed that ANS changed by the upright posture and postural tilt [13,14]. The ability to control blood pressure during a transition to upright posture in different individuals may be through the ANS mechanism. Upright posture may cause unloading of the arterial baroreceptors and vasoconstriction in the cutaneous circulation mediated by sympathetic activation measured by the microneurographic technique [15]. Baroreflex sensitivity (BRS), a cardiac autonomic function, was lower in the HUT positive group but was not the independent predictor of syncope [16]. HRV was used to access autonomic regulation during HUT in a previous study [17]. Our study used the simultaneous noninvasive recording of SKNA and ECG to estimate sympathetic tone and HRV during HUT. Thus, we can accurately and comprehensively record patients’ ANS in the four stages of HUT. Along with our finding that demonstrated the presyncope surge of SKNA, previous studies showed the increases in LF and reduction in HF of HRV prior to syncope [18] and the plasma adrenaline significantly increased from the prodrome to loss of consciousness [19]. In another study, the sympathetic tone assessed by HRV was elevated at the beginning of the test in VVS patients, which was compatible with our finding that rest phase SKNA is higher in the tilt-positive than the negative group [20]. In contrast to the consistently high SKNA in the tilt-positive group in our study, there are some controversial data in HRV data regarding the VVS patients [21]. Some fainters had increased, but others had decreased LFnu and LF/HF, the sympathetic components of HRV. The muscle sympathetic nerve activity (MSNA) was also used to access the autonomic regulation in VVS [22]. Consistent with our study, the MSNA increased during the early minutes of tilt, then vasovagal reaction occurred and was ushered in by abrupt cessation of MSNA. However, another study showed opposite results: the sympathetic assessed by MSNA and HR decreased but parasympathetic assessed by HRV increased 60 s before syncope onset [23].

### 4.2. Paradoxical Hemodynamic Response to Sympathetic Nerve Activity

G. Jacob et al. reported an interesting “paradoxical response” of vascular resistance to sympathetic stimulation. They found the dissociation between norepinephrine spillover and vascular responses to cold stress in lower limbs. A paradoxical decrease in local artery resistance despite increases in sympathetic activity by tyramine infusion was discovered [24]. The “paradoxical hemodynamic response” to sympathetic tone in our study shows the similar finding. We find an opposite linear relationship between BP, HR, and SKNA in the tilt-positive and tilt-negative groups. The “paradoxical hemodynamic response” only occurs in the provocation phase in the tilt-positive group and may be partially responsible for the syncope of the tilt-positive subjects. The possible mechanism might be related to elevated parasympathetic activity after nitroglycerin provocation, which overcame the sympathetic activity because it already burned out in the tilt-positive group but not in the tilt-negative group. This could be supported by elevated SDNN and declined LF/HF ratio during the provocation phase in our study, which are similar to a previous study [25].

### 4.3. Prediction of Syncope

Previously, Mallat et al. used the sustained increase in HR ≤ 18 beats per minute during the first 6 min of upright tilting to predict negative tilt tests [26]. Michela et al. demonstrated that using VLF at rest to predict the incidence of syncope during HUT (AUROC 0.889) [27]. Another algorithm utilized the age and BP changes during the early phase of HUT to predict the HUT result with positive and negative predictive values of 67.7 and 93% [28]. Recently, an algorithm combination of RR intervals, SBP trends, and their variability represented by LF power-generated cumulative risk was developed for VVS prediction with the sensitivity of 97.6% and specificity of 88.2% [10]. In our study, we use a novel index to predict the positive result of HUT. The “SKNA-SDNN index” has an AUROC 0.859, a sensitivity of 85%, and specificity of 83% to predict the positive result of HUT, which gives another choice to predict syncope. “SKNA-SDNN index” is composed of rest aSKNA and the changes of SDNN from resting to tilt-up and strengthens that both aSKNA and SDNN variation are important and should be integrated to predict the syncope.

### 4.4. Study Limitations

First, the sample size is small (41 patients). However, it is a novel study using the combination of SKNA and HRV to survey the autonomic regulation in VVS, and both SKNA and HRV are significantly different between tilt-positive and negative groups. Second, only 15 subjects have comprehensive recordings of corresponding SBP, DBP, and HR to analyze the linear relationship between them and aSKNA. We use multiple measurements in total 423 data points and generalized estimating equations model analysis to overcome the issue and show statistical significance. Third, since neuECG used traditional ECG electrodes to harvest electrical signals from the skin, the skin should be well prepared. The dirt, hair, oils, and desquamation of skin should be removed because they will affect the skin’s electrode contact. However, there is no absolute contraindication of recording neuECG. The environmental temperature and humidity might affect the sympathetic activity and should be controlled. We measured the SKNA on the average temperature of 23 ± 2 °C and moisture of 50 ± 5% in the recording area.

This study has several advantages. First, SKNA is a new technique, providing a better choice than solely HRV to evaluate the autonomic function in syncope patients during HUT. The SKNA can provide the real-time monitor of sympathetic tone during HUT. However, HRV takes at least 5 min of electrogram for analysis, which is difficult to correlate with the rapid change of BP and HR. Second, this is the first study to compare BP/HR parameters and SKNA in HUT. The paradoxical hemodynamic response to sympathetic nerve activity is found only at the provocation phase in the tilt-positive group; it also reflects the particular pattern of ANS regulation in tilt-positive patients. Third, the “SKNA-SDNN index” is a new and useful predictor for syncope in HUT, which gives high AUROC, sensitivity and specificity.

## 5. Conclusions

Higher SKNA at the rest phase is associated with positive HUT. The rest SKNA and SKNA-SDNN index are novel autonomic measurements that have the potential capability in predicting syncope in patients receiving HUT. The paradoxical hemodynamic responses to sympathetic drive might play an important role in VVS.

## Figures and Tables

**Table 1:** The baseline characteristics between tilt-negative and tilt-positive groups.

	Tilt-Negative		Tilt-Positive		*p* Value
	No.	(%)	No.	(%)	
**Participants**	25	(61.0)	16	(39.0)	
**Age (years)**	42.2 ± 14.0		46.6 ± 16.1		0.362
**Sex (female)**	17	(68.0)	12	(75.0)	0.631
**DM**	4	(16.0)	2	(12.5)	0.757
**Hypertension**	4	(16.0)	4	(25.0)	0.478
**CVA**	1	(4.0)	0	(0)	0.418
**HBV**	3	(12.0)	3	(18.8)	0.551
**VT**	1	(4.0)	0	(0)	0.418
**AF**	1	(4.0)	0	(0)	0.418
**COPD**	2	(8.0)	0	(0)	0.246
**MVP**	3	(12.0)	2	(12.5)	0.962
**LVEF (%)**	70.7 ± 5.7		68.2 ± 8.7		0.348

AF, atrial fibrillation; CI, confidence interval; COPD: chronic obstructive pulmonary disease; CVA, cerebrovascular accident; DM, diabetes mellitus; HBV, hepatitis B carrier; LVEF: left ventricular ejection fraction; MVP, mitral valve prolapse; VT: ventricular tachycardia. *p*-value was obtained by *t* test for continuous variables and by Pearson’s chi-square test for categorical variables.

**Table 2:** The difference of BP/HR and aSKNA between tilt-negative and tilt-positive groups in different phases.

	Phase	Tilt-Negative		Tilt-Positive		*p* Value
		Mean	SD	Mean	SD	
**Rest**	**SBP (mmHg)**	117.9	15.7	122.9	18.8	0.355
	**DBP (mmHg)**	75.4	15.7	75.6	9.5	0.970
	**MBP (mmHg)**	89.6	11.7	91.4	12.0	0.636
	**HR (beats/min)**	67.2	6.9	66.4	8.0	0.733
	**aSKNA (** **μ** **V)**	1.02	0.29	1.21	0.27	0.034 *
**Tilt-up**	**SBP (mmHg)**	118.4	13.4	118.2	15.9	0.952
	**MBP (mmHg)**	76.1	9.9	77.5	9.9	0.678
	**DBP (mmHg)**	90.2	10.6	91.0	11.3	0.823
	**HR (beats/min)**	75.3	7.7	77.3	12.2	0.517
	**aSKNA (** **μ** **V)**	1.29	0.34	1.35	0.30	0.538
**Provocation**	**SBP (mmHg)**	109.9	12.5	101.2	13.1	0.040 *
	**DBP (mmHg)**	69.8	9.6	66.5	9.8	0.300
	**MBP (mmHg)**	83.2	10.2	78.1	10.6	0.136
	**HR (beats/min)**	89.9	11.9	84.0	14.5	0.166
	**aSKNA (** **μ** **V)**	1.23	0.35	1.39	0.35	0.163
**Recovery**	**SBP (mmHg)**	111.2	15.7	108.8	14.3	0.613
	**DBP (mmHg)**	68.4	11.2	66.8	10.0	0.642
	**MBP (mmHg)**	82.7	9.4	80.8	11.2	0.562
	**HR (beats/min)**	69.8	10.7	60.2	6.3	0.003 *
	**aSKNA (** **μ** **V)**	1.18	0.32	1.27	0.39	0.386
**Tilt-up/Rest**	**HR**	1.12	0.10	1.17	0.13	0.254
	**aSKNA**	1.30	0.29	1.13	0.18	0.038 *
**Provocation/Rest**	**HR**	1.34	0.16	1.27	0.17	0.156
	**aSKNA**	1.24	0.31	1.16	0.22	0.351
**Provocation/Tilt-up**	**HR**	1.19	0.07	1.09	0.10	<0.001 *
	**aSKNA**	0.96	0.10	1.04	0.21	0.082

aSKNA, average skin sympathetic nerve activity; BP, blood pressure; DBP, mean diastolic blood pressure; HR, mean heart rate; MBP, mean blood pressure; SBP, mean systolic blood pressure; SD, standard deviation. * *p* < 0.05 was obtained by *t* test.

**Table 3:** The effect of aSKNA (μV) on BP/HR in different phases, stratified by the tilting-test result.

		Tilt-Negative (*n* = 7)					Tilt-Positive (*n* = 8)					*p* for Difference in Adj. β ^d^
Phase	Outcomes	t ^a^	Mean t ^b^	Adj. β ^c^	SE	*p* Value	t ^a^	Mean t ^b^	Adj. β ^c^	SE	*p* Value	
**Rest**	**SBP (mmHg)**	28	4	5.48	5.42	0.311	32	4	11.39	3.84	0.003 *	0.130
	**DBP (mmHg)**			3.18	3.28	0.334			2.76	3.52	0.433	0.639
	**MBP (mmHg)**			3.96	3.49	0.256			1.00	2.86	0.726	0.921
	**HR (bpm)**			3.55	4.47	0.427			6.73	2.18	0.002 *	0.434
**Tilt-up**	**SBP (mmHg)**	70	10	2.03	3.24	0.530	80	10	1.27	3.15	0.686	0.834
	**DBP (mmHg)**			8.56	3.85	0.026 *			1.50	2.45	0.541	0.340
	**MBP (mmHg)**			7.28	3.08	0.018 *			1.35	2.10	0.520	0.344
	**HR (bpm)**			3.17	4.04	0.433			−0.08	2.56	0.974	0.462
**Provocation**	**SBP (mmHg)**	98	14	19.32	3.50	<0.001 *	58	7.3	−11.65	5.08	0.022 *	0.038 *
	**DBP (mmHg)**			7.31	3.95	0.064			−13.75	3.82	<0.001 *	0.016 *
	**MBP (mmHg)**			11.00	3.62	0.002 *			−11.39	3.94	0.004 *	0.026 *
	**HR (bpm)**			27.64	5.46	<0.001 *			−3.57	4.36	0.413	<0.001 *
**Recovery**	**SBP (mmHg)**	21	3	11.80	5.61	0.035 *	36	4.5	1.71	8.23	0.835	0.784
	**DBP (mmHg)**			8.58	4.99	0.086			11.79	3.75	0.002 *	0.351
	**MBP (mmHg)**			8.07	4.32	0.062			5.10	4.75	0.282	0.696
	**HR (bpm)**			8.17	7.83	0.296			4.99	4.26	0.242	0.705

Adj. β, adjusted mean; aSKNA, average skin sympathetic nerve activity; bpm, beats per minute; BP, blood pressure; DBP, diastolic blood pressure; HR, heart rate; MBP, mean blood pressure; SBP, systolic blood pressure; SE, standard error. ^a^ The t is the total amount of time points in this group. ^b^ The mean t is mean amount of time points per patient in this group. ^c^ All β coefficients were obtained from the generalized estimating equations model with an exchangeable correlation structure adjusted for age and sex. Only tilt-positive (*n* = 8) and tilt-negative (*n* = 7) in this table. ^d^ *p* for the difference was the difference of adjusted β coefficients between tilt-positive and tilt-negative groups. * *p* < 0.05.

**Table 4:** Heart rate variability between tilt-negative and tilt-positive groups.

	Phase	Tilt-Negative		Tilt-Positive		*p* Value
		Median	Q1–Q3	Median	Q1–Q3	
**Rest**	**SDNN (ms)**	35.6	26.3–65.5	31.8	26.6–36.9	0.415
	**RMSSD (ms)**	29.3	12.0–61.8	19.6	12.7–27.2	0.264
	**LFnu (%)**	43.7	34.0–71.1	58.6	45.1–69.9	0.389
	**LF/HF**	0.78	0.51–2.48	1.41	0.82–2.33	0.389
**Tilt up**	**SDNN (ms)**	37.2	24.3–50.6	40.2	35.8–52.4	0.775
	**RMSSD (ms)**	22.8	10.9–35.1	17.5	14.5–28.7	0.668
	**LFnu (%)**	61.0	45.0–72.4	67.1	61.0–74.6	0.574
	**LF/HF**	1.56	0.82–2.65	2.04	1.56–2.94	0.574
**Provocation**	**SDNN (ms)**	66.5	54.6–99.3	99.8	70.2–158.1	0.074
	**RMSSD (ms)**	22.4	9.5–37.2	31.3	21.4–52.1	0.496
	**LFnu (%)**	79.7	48.6–88.8	65.3	47.5–69.9	0.070
	**LF/HF**	3.93	0.95–7.92	1.88	0.91–2.32	0.070
**Recovery**	**SDNN (ms)**	53.4	27.6–89.6	61.5	52.7–62.4	0.238
	**RMSSD (ms)**	51.1	13.0–83.4	43.8	24.4–48.7	0.483
	**LFnu (%)**	53.8	33.9–66.8	61.5	55.6–64.1	0.164
	**LF/HF**	1.17	0.51–2.01	1.60	1.25–1.78	0.164
**Tilt-up/Rest**	**SDNN**	0.87	0.75–1.09	1.24	1.08–1.33	0.002 *
	**RMSSD**	0.72	0.57–0.85	0.81	0.67–1.03	0.442
	**LFnu**	1.28	0.98–1.55	1.10	0.92–1.58	0.364
	**LF/HF**	1.85	0.97–2.72	1.28	0.83–2.64	0.745
**Provocation/Rest**	**SDNN**	1.91	1.06–2.43	3.08	2.33–5.77	0.020 *
	**RMSSD**	0.59	0.37–0.88	1.70	0.60–2.82	0.042 *
	**LFnu**	1.41	1.15–1.95	0.94	0.78–1.38	0.042 *
	**LF/HF**	2.88	1.59–6.06	0.81	0.45–1.86	0.021 *
**Provocation/Tilt-up**	**SDNN**	1.78	1.18–2.45	1.85	1.75–4.42	0.415
	**RMSSD**	0.80	0.61–1.46	1.26	0.69–4.38	0.234
	**LFnu**	1.09	1.01–1.30	1.02	0.64–1.31	0.385
	**LF/HF**	1.36	1.02–2.73	1.05	0.31–1.71	0.245

LF/HF, low frequency to high frequency ratio; LFnu, low frequency in normal units; Q1, 25th percentile; Q3, 75th percentile; SDNN, standard deviation of all normal-to-normal intervals; RMSSD, the square root of the mean of the sum of the squares of differences between adjacent NN intervals. * *p* < 0.05 by Mann-Whitney U Test.

## Data Availability

The data presented in this study are available upon request from the corresponding author. The data are not publicly available due to ethical reasons.
